# Antibody titres and boosting after natural malaria infection in BK-SE36 vaccine responders during a follow-up study in Uganda

**DOI:** 10.1038/srep34363

**Published:** 2016-10-05

**Authors:** Masanori Yagi, Nirianne M. Q. Palacpac, Kazuya Ito, Yuko Oishi, Sawako Itagaki, Betty Balikagala, Edward H. Ntege, Adoke Yeka, Bernard N. Kanoi, Osbert Katuro, Hiroki Shirai, Wakaba Fukushima, Yoshio Hirota, Thomas G. Egwang, Toshihiro Horii

**Affiliations:** 1 Department of Molecular Protozoology, Research Institute for Microbial Diseases, Osaka University, 3-1 Yamadaoka, Suita, Osaka 565-0871 Japan; 2 Department of Public Health, Faculty of Medicine, Osaka City University, Osaka 545-8585, Japan; 3 Sumida Hospital, Medical Co. Living Together Association (LTA) Clinical Pharmacology Center, Tokyo 130-0021 Japan; 4 Med Biotech Laboratories, Plot 4-6 Bell Close, Port Bell Road Luzira, Kampala, Uganda; 5 Department of Disease Control and Environmental Health, School of Public Health, College of Health Sciences, Makerere University, P.O. Box 7072, Kampala, Uganda; 6 The Research Foundation for Microbial Diseases of Osaka University, 2-9-41 Yahata-cho, Kanonji, Kagawa 768-0061 Japan

## Abstract

The malaria vaccine BK-SE36 is a recombinant protein (SE36) based on the Honduras 1 serine repeat antigen-5 of *Plasmodium falciparum*, adsorbed to aluminium hydroxide gel. The phase Ib trial in Uganda demonstrated the safety and immunogenicity of BK-SE36. Ancillary analysis in the follow-up study of 6–20 year-old volunteers suggest significant differences in time to first episodes of clinical malaria in vaccinees compared to placebo/control group. Here, we aimed to get further insights into the association of anti-SE36 antibody titres and natural *P. falciparum* infection. Children who received BK-SE36 and whose antibody titres against SE36 increased by ≥1.92-fold after vaccination were categorised as responders. Most responders did not have or only had a single episode of natural *P. falciparum* infection. Notably, responders who did not experience infection had relatively high anti-SE36 antibody titres post-second vaccination compared to those who were infected. The anti-SE36 antibody titres of the responders who experienced malaria were boosted after infection and they had lower risk of reinfection. These findings show that anti-SE36 antibody titres induced by BK-SE36 vaccination offered protection against malaria. The vaccine is now being evaluated in a phase Ib trial in children less than 5 years old.

Malaria is widespread in tropical and subtropical regions and despite substantial progress in malaria control, millions of people, particularly in Africa, remain at risk of disease and death^1^. RTS, S, a *P. falciparum* pre-erythrocytic vaccine, was the first malaria vaccine given a positive scientific opinion by European Medicines Agency (EMA) Committee for Medicinal Products for Human Use^2^. However, vaccine efficacy figures are below anticipated goals with only moderate to modest efficacy^3^,^4^,^5^ and constrained by allele-specific immunological protection in 5–17 month-old children^6^. Additionally, some safety concerns resulted in recommendation for pilot implementation before further rollout^7^. An asexual blood-stage malaria vaccine that prevents disease, thus, remains highly desirable.

To date, no blood-stage vaccine candidate has been evaluated in a phase III trial^8^. Majority of those that completed either phase IIa or phase IIb trials are merozoite surface antigens. Apical membrane antigen 1 (PfAMA1), merozoite surface protein 1 (PfMSP1) and Combination B (MSP1, MSP2 and RESA [ring stage erythrocyte surface antigen]) were not effective to prevent clinical malaria in phase II trials but did show strain-specific reduction in malaria infections (PfAMA1, Combination B) or delayed significantly the length of the prepatent period after sporozoite challenge (PfMSP1) (reviewed in refs 8, 9, 10). A 3D7 based adjuvanted AMA1 (FMP2.1/AS02A) had efficacy against clinical malaria for the homologous parasite (64.3%) but only 17.4% efficacy overall in a phase II study^11^. MSP3 long synthetic peptide with aluminum hydroxide adjuvant showed clinical protection in a phase I study (incidence rates of 1.2 [15 μg dose] and 1.9 [30 μg dose] cases of ≥5,000 parasites/μL per 100 days compared to 5.3 cases for the control^12^); but a multicenter phase II trial for GMZ2/Alum (combination of glutamate-rich protein [PfGLURP] and MSP3) reported a low protective efficacy (age and site-adjusted per-protocol analysis of vaccine efficacy was 13.6% [95% CI, 3.6–23%]; efficacy against severe malaria = 27% [95% CI, −44–63%]^13^ that does not justify further clinical development of the same vaccine formulation. Several novel candidates are also being explored (e.g. non-merozoite surface antigens trophozoite exported protein 1, Tex1; schizont egress antigen-1, SEA-1, etc.) and recent efforts are geared for second generation multi-allele (e.g. iterations of MSP1, erythrocyte binding antigens [EBA-175] or evaluation of ‘diversity covering’ [DiCo] strategy for AMA1), multi-antigen vaccines with improved antibody delivery platforms (e.g. use of virus like particles for reticulocyte-binding protein homolog 5 [RH5]; epitope specific approaches) for possible combination of blood-stage antigens towards development of multi-stage malaria vaccine^8^,^9^,^10^.

*Plasmodium falciparum* serine repeat antigen 5 (*Pf*SERA5) is a blood stage antigen abundantly expressed on the merozoite surface^14^,^15^. The 120 kDa protein belongs to the SERA protein family and is processed into 47, 50, 6, and 18 kDa domains upon schizont rupture. Growth inhibition and antibody dependent cellular inhibition assays with antibody against the N-terminal domain demonstrated the antigen’s capacity to limit red blood cell invasion and/or growth *in vitro*^16^,^17^,^18^,^19^. In epidemiological studies, correlation between high anti-N-terminal domain antibody levels and low fever and reduced parasitemia^14^,^20^,^21^; as well as limited polymorphism of *Pf*SERA5^22^ suggest that this antigen is a promising vaccine candidate. SE36, the recombinant N-terminal domain of *Pf*SERA5 without polyserine repeats was expressed in *Escherichia coli* and formulated with aluminium hydroxide gel as BK-SE36^14^,^15^.

We conducted phase Ia clinical trial in Japan and phase Ib clinical trial in Uganda for BK-SE36^14^,^23^. In both trials the vaccine was deemed safe and had acceptable reactogenicity. All malaria naïve healthy Japanese adults who received BK-SE36 responded to the vaccine resulting in 100% seroconversion^14^. In the phase Ib Ugandan trial, pre-existing anti-SE36 antibody, acquired as a result of natural infection, influenced seroconversion especially in older cohorts^23^. Those who received full dose BK-SE36 had a higher antibody response than those vaccinated with half dose, but only those in the younger age cohorts (6–10 year-old children) had greater than 2-fold increase in antibody titre. The precise mechanism of how antibody titres modulate immune responses remains unclear. Suppressed immune response (either through immune tolerance or immune ignorance) was also observed in semi-immune adults with high baseline antibody titres in the MSP3 phase Ib trials in Burkina Faso^24^,^25^. During the follow-up/longitudinal study after the phase Ib trial, it was observed that BK-SE36 afforded some protection against symptomatic malaria^23^. In order to gain further insights into the immune response to BK-SE36, we correlated changes in anti-SE36 antibody titres and observed malaria incidences during the one year longitudinal study post-second vaccination.

Assessing the persistence of vaccine-induced immune response and potential boosting by natural infection is important to understand the acquisition, maintenance and longevity of the immune response; however, much of the studies on antibody maintenance and boosting as a result of natural infection were observational with only a handful carried out in vaccinees (Supplemental Table S1). With regular/monthly blood samplings for anti-SE36 antibody titres coupled with monitoring for malaria infection throughout the follow-up period, we were able to see the dynamics in antibody titre changes in our volunteers. A blood-stage vaccine should be able to reduce disease episode, *i.e.,* infections and symptoms may occur in vaccinees but less often and/or with less severity. Results reported here provide further proof that BK-SE36 can reduce parasitemia and subsequent clinical malaria; and that the antibody response in BK-SE36 vaccine responders can be boosted by natural malaria infection.

## Results

### Categorising response to BK-SE36 vaccination

**Figure 1:**
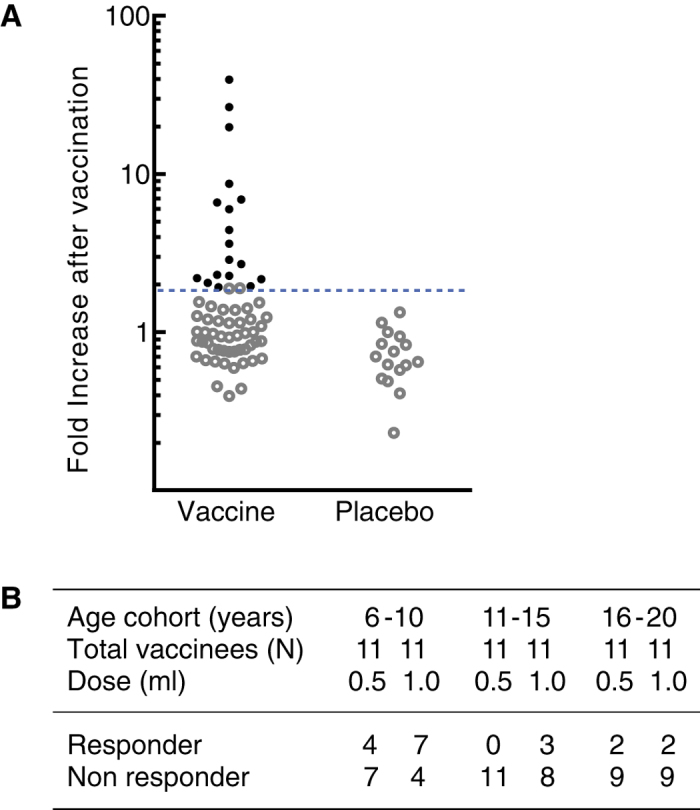
Distribution of the fold increase in antibody titres after two vaccinations. (**A**) Filled circles (●) and open circles (○) denote individual volunteers designated as responders or non responders, respectively in the vaccine and placebo groups. Dotted line represents the cutoff level in fold increase of antibody titre after vaccination for grouping according to immune response based on the upper limit of the 99% CI of fold increase in the placebo group. (**B**) The table summarises the proportion of vaccinees designated as responder and non responder for each age cohort and vaccine dose [half- (0.5 ml) or full-dose (1.0 ml)].

In the trial volunteers aged 6–20 years-old, antibody responses to BK-SE36 vaccination can be differentiated into two: one group of vaccinees that had considerable antibody response, and the other group of vaccinees that did not have an obvious increase in their anti-SE36 antibody titres 21 days post-second vaccination. When categorizing the effect of vaccination on antibody response, the ELISA data for antibody titres on Day 0 and 42 were used^23^. The distribution of the resultant fold increase in antibody titres in vaccinees and placebo is shown in Fig. 1A. The vaccinated group had up to 39.5-fold increase while the maximum fold increase in the placebo group was 1.51. At a cutoff point which is derived from the upper bound of the 99% confidence interval (CI) of the fold increase in the placebo group, vaccinees were broadly classified as responders (≥1.92-fold increase in antibody titre) or non responders (<1.92-fold increase). 99% CI was calculated as average fold increase ± 2.58× standard deviation of logarithmic-converted fold increase values. Notably, more than half of the vaccinees in the youngest cohort are responders (Fig. 1B). Children aged 6–10 years old that received full dose of BK-SE36 (1 ml) had higher fold increase in antibody titre compared to half-dose group (0.5 ml) (Fig. 1B), suggesting a dose-response effect.

### Antibody dynamics in responders during natural infection

To address the question of whether the antibody response to BK-SE36 can be boosted by natural infection/reinfection, we applied the following analyses: (i) when parasitemia ≥100 parasites/μl blood was observed at a scheduled visit, antibody titres one month before and one month after the recorded infection were compared; (ii) when parasitemia ≥100 parasites/μl blood was observed during an unscheduled visit, antibody titres at the nearest monthly visit before infection and the succeeding monthly visit after infection were compared. The blood-stage parasitemia threshold (≥100/μl) to define infection was based on the sensitivity limit for malaria detection in the field using microscopy^26^,^27^. Parasite infection(s) that recurred within 28 days were counted as one infection. At the end of the follow-up period (Day 365), no child had malaria infection but there were those who experienced infections within 28 days prior to the end of the follow-up (n = 24) and, thus, to monitor antibody titre changes, titres one month following Day 365 were used for comparison.

**Figure 2:**
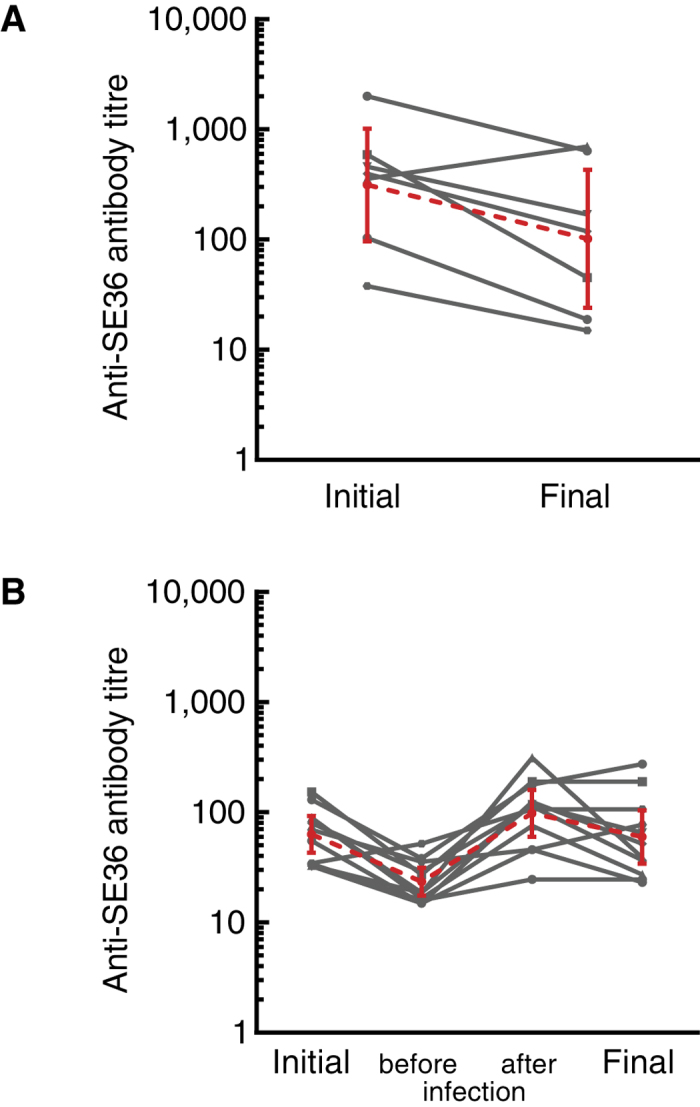
Antibody titre changes in vaccine responders that had (**A**) no or (**B**) at least one *P. falciparum* infection during Day 130–365. Initial, before, after and final denote baseline antibody titres obtained 21 days post-second vaccination, titre at a monthly visit before the observed malaria infection, titre at a monthly visit after malaria infection, and titre at 365 days post-second vaccination, respectively. Lines and markers in red show GM of antibody titres with 95% CI. (**A**) GM = 314.0 (95% CI, 96.64–1020) and GM = 102.0 (95% CI, 24.16–430.4) for Initial and Final, respectively. (**B**) GM = 62.8 (95% CI, 42.86–92.01); GM = 20.88 (95% CI, 16.38–26.63); GM = 97.29 (95% CI, 59.46–159.2) and GM = 54.19 (95% CI, 31.47–93.34) for Initial, before infection, after infection and Final, respectively.

Dynamics of antibody titre changes in responders that did and did not experience infection are compared in Fig. 2. Generally a responder that did not experience malaria infection had a high geometric mean (GM) antibody titre 21 days post-second vaccination (314 units; 95% CI, 96.64–1020.0). Titres gradually waned by the end of the follow-up, although there was one responder that showed an increase in antibody titre from 130–365 days post-second vaccination but had no record of malaria infection (Fig. 2A). This child may have experienced asymptomatic infection or had parasitemia below the detection limit for microscopy. By Day 365, GM of the antibody titres in the responder group that did not experience infection was 102 units (95% CI, 24.16–430.4).

For the responder group that experienced malaria infection, GM of antibody titres at the start of the follow-up period was 62.8 units (95% CI, 42.86–92.01) (Fig 2B). Antibody titres further declined until such time when these volunteers experienced malaria infection (GM_before infection_ = 20.88 units; 95% CI, 16.38–26.63). However, after the first malaria infection, the antibody titres in this group of vaccinees increased to GM = 97.29 units (95% CI, 59.46–159.2). Nine of 11 children did not experience a second malaria infection until Day 365, suggesting that they were protected from further infection. By Day 365, GM of the antibody titres in the responder subpopulation that experienced infection was 54.19 units (95% CI, 31.47–93.34) (Fig. 2B).

Supplemental Fig. S1 show the individual anti-SE36 antibody changes before and after malaria infection in responders, non responders and control. Most responders have greater than 4-fold increase in GM after infection compared to non responders and control (Responder GM_before infection_ = 21.61 [95% CI, 17.35–26.91] *vs* Responder GM_after infection_ = 97.79 [95% CI, 60.82–157.1] resulting to 4.5-fold increase; Non responder GM_before infection_ = 68.35 [95% CI, 45.88–101.8] *vs* Non responder GM_after infection_ = 99.88 [95% CI, 69.51–143.5], 1.46-fold increase; Control GM_before infection_ = 63.47 [95% CI, 46.61–86.42] *vs* Control GM_after infection_ = 85.93 [95% CI, 61.93–119.2], 1.35-fold increase).

### Fold increase in antibody titres after infection

Factors that may influence antibody titre changes after infection in responder, non responder and control groups were determined. The dataset for comparison of fold increase in antibody titres post infection used all data from volunteers that experienced at least one *P. falciparum* infection during the follow-up period (Supplemental Fig. S1). Odds ratio was used to look for the association of fold increase in antibody titres after the first infection to responder status, age, GM antibody titre before infection and parasitemia level during the first infection (Supplemental Table S2). Crude and adjusted odds ratio for children whose antibody titre increased by more than 3.35-fold post infection showed that the considerably high fold increases in antibody titres post-infection were only obtained in BK-SE36 vaccinees and more so in the responder group. The 3.35 threshold was the 75^th^ percentile fold increase in antibody titre after first infection in infected volunteers.

This observation was robustly supported by looking at the GM of fold increases after first infection. GMs were significantly different among responder, non responder and placebo/control, following adjustment for age, antibody titre before first infection and parasitemia during infection (p = 0.005, Table 1). In responders, the GM of the fold increase in antibody titre after the first infection significantly increased by 3.3-fold (95% CI, 2.1–5.3) from the baseline (before infection). This 3.3-fold increase was 1.5 times higher than that observed in the non responder group (95% CI, 0.9–2.6), and 2.3 times higher than in placebo/control group (95% CI, 1.3–3.8). Antibody titre levels before the first infection had no significant association with fold increases in antibody titres after infection (p = 0.156). Age (p = 0.175) and parasitemia (p = 0.091) did not appear to be a major influence for the degree of fold increase in antibody titres before and after infection. Thus, Table 1 and Supplemental Table S2 show that the anti-SE36 antibody level induced by BK-SE36 vaccination is significant and independently associated with fold increases after the first infection.

### Risk for reinfection

From among 77 children who had at least one infection, 36 volunteers were infected more than once. The proportion of children that experienced more than one infection from the responder group (2/11; 18%) was in contrast to the number that experienced reinfection in non responders (12/26; 46%) and control (22/40; 55%). In Table 2, responders had decreased odds to *P. falciparum* reinfection (0.2; 95% CI, 0.03–0.8), compared with non responders. Following adjustment for age, antibody titre after first infection and parasitemia, the corresponding odds ratio for reinfection in the responder group was 0.1 (95% CI, 0.01–0.5). Thus, the observed association of fewer reinfections in responders was robust and not influenced by age, antibody titre or parasitemia levels.

## Discussion

Besides safety and immunogenicity of BK-SE36^23^, a follow-up study was also conducted in 6–20 years-old to be able to compare malaria incidences between BK-SE36 vaccinees and the control group 130–365 days post-second vaccination. We have previously shown that the immune response against BK-SE36 was higher in the youngest cohort (6–10 years-old) and that BK-SE36 confers some protection in follow-up volunteers. Here we looked in detail at the association of antibody response profiles in BK-SE36 vaccinees and malaria infection episodes.

Vaccine responders have significant fold increases in antibody titres after two administrations of BK-SE36. For responders who did not experience any *P. falciparum* infection during the follow-up period, their antibody titre levels were relatively high post-second vaccination. Analysis also indicates that their anti-SE36 antibody titres were significantly associated with protection against malaria infection. For responders who experienced infection, all of them had significant fold increases after vaccination but their relative antibody levels remained low. These children had titers that waned to much lower levels especially at a visit prior to recorded malaria infections. However, upon natural infection majority had more than 3.35-fold increase in antibody titre, suggesting the presence of immunological memory. Moreover, most of these children experienced only a single infection. It can be argued that fold increase only occurs when antibody titres are low prior to infection. As shown in Table 1 and Supplementary Table S2, boosting is not biased by baseline antibody titres and that even with low antibody titres only a small proportion of children in the non responder (31%) and control group (10%) showed more than 3.35-fold increase in antibody titre. These responses show that natural boosting also occurred in both non responders and control group, but to a lesser extent (also see Supplementary Fig. S1). Fewer evidences of boosting in non responders and the control group may reflect the inherent low immunogenicity or acquisition of SE36 as observed in previous sero-epidemiological studies in malaria holo-endemic areas^14^.

Boosting of the immune response in BK-SE36 vaccinees was similarly observed in challenge infection studies using squirrel monkeys^14^,^19^. Male squirrel monkeys immunised with BK-SE36 and challenged with homologous parasite (*P. falciparum* Indochina-1/CDC strain) had lower parasitemia than control monkeys and showed booster effects on antibody titres after infection in contrast to control monkeys whose anti-SE36 antibody titre was not induced even after challenged infection. In the follow-up study, although there were variations or between-person heterogeneity in vaccine efficacy and some children in the non responder and control group had increases in antibody titre post infection, only the responder group had lower number of observed *P. falciparum* reinfections. Thus, in BK-SE36 responders, natural infection can boost the immune response and this host immunity can be expected to suppress subsequent parasite growth that can lead to prevention or reduction in severe illness.

It remains to be elucidated whether induction of cell-mediated responses, aside from antibody responses, correlate with the observed protection. RTS, S, for example, induces high levels of anti-CSP antibody titres and CSP-specific CD4^+^ T cells and both have been correlated with protection^28^. Also functional antibody studies will help to dissect further the association of anti-SE36 antibody titre and protection from malaria.

In the follow-up study, for ethical reasons, even asymptomatic children were cleared of any existing parasitemia using standard antimalarial drugs when infection was detected by microscopy, thus we were unable to examine antibody fluctuations relative to change in parasite density or length of infection. But based on the analysis, parasitemia does not appear to be a major influence for the degree of fold increase in antibody titre before and after infection. All the same, anti-SE36 antibody level induced by BK-SE36 vaccination is significantly protective against malaria infection and the induced antibody titres can be boosted by intermittent *P. falciparum* infections in the field. That some responders did not experience infection for a year suggest that it may be possible to have a higher protective efficacy if immune response can be further enhanced. Also based on these results, a double-blind trial for young children below five, who are expected to respond well to BK-SE36 vaccination, is underway.

## Materials and Methods

### Clinical trial and follow-up study

Serum samples and data of malaria incidence/parasitemia were obtained from stage 2 BK-SE36 Phase Ib clinical trial and follow-up study volunteers^23^. Ethical approvals for the trial, longitudinal study, and for blood samples to be taken and stored for use in future studies were obtained from Osaka University Research Ethical Review Board (Japan; RIMD 20-3; OU: 287), Research Foundation for Microbial Diseases of Osaka University Ethical Committee (BIKEN-IRC) (Japan), Med Biotech Laboratories Institutional Review Committee (MBL-IRC: IRB-00003990-MBL-BIOMEDICAL, IRB-00003995-MBL-BIOMEDICAL) (Uganda), and Uganda National Council for Science and Technology (UNCST HS 635, HS 866)^23^. Written informed consents were obtained from all volunteers (or in the case of children, their parents and/or guardians) before screening and enrolment. The study was conducted in accordance with approved protocols and regulations. Details of both the clinical trial and follow-up study have been described previously^23^.

Briefly, stage 2 of the clinical trial was conducted in healthy children and young adults aged 6–20 years (n = 84) after demonstration of the safety of BK-SE36 in stage 1 Ugandan adults (≥21 years-old). Three age cohorts (6–10, 11–15 and 16–20 years old) were randomised to receive either 0.5 or 1.0 ml of BK-SE36 or saline. In each age group, 22 received BK-SE36 (n = 11 for 1.0 ml or full-dose and n = 11 for 0.5 ml or half-dose) and 6 received saline (3 volunteer for 1.0 ml and 3 for 0.5 ml), for a total of 66 participants in the BK-SE36 group and 18 in the placebo group. Subcutaneous administration was done twice at 21-day intervals and antibody titres were measured for the serum samples collected on Day 0 (before 1^st^ vaccination), Day 21 (before 2^nd^ vaccination) and Day 42 (21 days after 2^nd^ vaccination). In the follow-up study (130–365 days post-second vaccination), to increase statistical power, 50 additional control children (age-, gender-, and as much as possible, locality-matched) were recruited and together with vaccinees had blood samples obtained monthly (28-day interval for active surveillance) or whenever sick (passive surveillance). The additional control group was evaluated in a screening activity that used the same inclusion and exclusion criteria as in the clinical trial. On a scheduled day matched vaccinee and control volunteer underwent the same routine assessments for vital signs, physical examination, monthly questionnaire and blood smear. Two vaccinees that received saline during the clinical trial were not included in the longitudinal study because of pregnancy and one had moved out of the study area.

### Enzyme-Linked Immunosorbent Assay

Anti-SE36 antibody titres during the 130–365 days post-second vaccination were determined from anonymised serum samples using the standard procedure for enzyme-linked immunosorbent assay (ELISA)^14^,^23^. Briefly, 0.3 μg SE36 protein was coated on each 96-well flat-bottomed Nunc-Immuno plates (Nunc, Roskilde, Denmark). After coating overnight at 4 °C, plates were washed with PBS containing 0.05% Tween 20 (PBS/T) and blocked for an hour with 5% skim milk in PBS at 37 °C. Plates were again washed three times with PBS/T prior to addition of serum samples for 1 h at 37 °C. A serum pool from 10 Ugandan individuals with high anti-SE36 antibody titres was used as a standard and a malaria naïve Japanese serum was used as a negative control. Starting from a 100-fold dilution, serum samples from the follow-up study were added to eight wells in two-fold serial dilutions. After washing with PBS/T, horseradish peroxidase-conjugated anti-human IgG antibody (55220; Cappel ICN Pharmaceuticals Inc, Aurora, OH) diluted 1∶2,000 in 5% skim milk in PBS/T was added to the plates and incubated at 37 °C for another hour. Thereafter, plates were washed and incubated with 100 μl freshly prepared citrate-phosphate buffer (pH 5.0) containing 0.2% hydrogen peroxide and OPD tablet (154-01673; Sigma-Aldrich Corp., St. Louis, MO) for 15 minutes. The reaction was stopped with 100 μl of 2 M sulfuric acid and optical density was read at 492 nm. The high titer serum pool from 10 Ugandan individuals was used to generate a standard curve on each ELISA plate. A 5,000 unit value was assigned to this standard serum, derived from the reciprocal of its dilution where an absorbance of 1.0 was obtained at OD_492_. ELISA units of the test sera were calculated by comparing with the standard serum using a parallel line assay (Bioassay Assist software ver. 2.0.7).

### Booster effect analysis

To look for correlations on antibody titre changes before and after natural infection, the start of the follow-up period (130 days post-second vaccination, when we had equal numbers of vaccinees and controls) was taken as the initial timepoint. Monthly titres were analysed for all volunteers. For boosting analyses, all titres <15 units (relative to the 5,000 unit of the Ugandan standard) were assigned a value of 15 to avoid bias or misinterpretation for a significant antibody titre increase when the comparison/baseline titre is very low. The threshold value of 15 units was set as the lowest possible antibody titre based on the average of 104 independent measurements using the Japanese negative control sera plus 3x standard deviation.

### Definition of blood-stage infection

Parasitemia was recorded on the same day using thick and thin blood smears. Parasite count assumed a fixed white blood cell (WBC) count of 8,000/μl and was expressed as the number of asexual parasites per microliter of blood. Volunteers that were blood smear positive during clinic visits were treated with antimalarial drugs (artemether-lumefantrine or dihydroartemisinin-piperaquine) as detailed in the protocol^23^. A slide was declared negative if no asexual parasites were found after counting 500 WBCs (gametocytes were found once during the follow-up period in 5 volunteers but these were not considered for further analysis because no count was recorded). Consecutive malaria episodes that occurred within 28 days were regarded as a relapse and counted as one infection.

Since blood-stage vaccines may act as disease-ameliorating rather than preventing infection itself; and because in a semi-immune population, submicroscopic blood-stage infections are common, the definition of malaria infection in this study was refined to ≥100 parasites/μl blood by microscopy. One hundred parasites per μL blood was regarded as the detection capability of a typical microscopist under general field conditions^26^,^27^. This lower limit of detection for *P. falciparum* infection, is also regarded to have the sensitivity to detect symptomatic infections in non-immune individuals^29^. The total number of observed infection with ≥100 parasites/μl during the period of March-November 2011 was 77.

### Statistical analysis

To analyse the threshold to define the category of antibody response to vaccination, the dataset consists of 84 children (66 BK-SE36 vaccinees and 18 placebo group). Geometric means (GMs) and 95% CIs of antibody titres were computed. These statistical analyses were performed using GraphPad Prism 6 (GraphPad Software; La Jolla, CA).

To analyse fold increase in antibody titres after natural infection (boosting), the dataset consist of 77 children. The 77 is selected from 132 volunteers (66 vaccinees, 16 placebo, 50 additional controls) who had at least one infection during the follow-up period. Multivariate linear regression was used to estimate GMs of the fold increase after the first infection according to response categories (responder, nonresponder and control), adjusting for the effect of age (6–10 or 11–60 years old), antibody titre levels before infection (<52 or ≥52 units) and parasitemia during the first infection (<5,000 and ≥5,000 parasites/μl blood). The designated 52 units threshold for the aforementioned category represents the maximum antibody titer before first infection in responders. Parasitemia at 5,000 parasites/μl is generally regarded as indicator of risk for clinical malaria in the study site^23^. The ratios of GMs and the 95% CIs between pairwise response categories were obtained. The significant difference among categories for each model variable was tested by F-test. In addition, the odds ratios of the 3.35-fold increase in antibody titre after the first infection for response categories were estimated with multivariate logistic regression adjusting for the same three factors. The 3.35 threshold represents the 75^th^ percentile for the antibody response after first infection in infected volunteers. The odds ratio of reinfection for response categories were estimated with multivariate logistic regression adjusting the effect of age, antibody titer levels after the first infection (based on median antibody titres) and parasitemia during the first infection. Statistical analyses were performed using SAS 9.3 (SAS Institute Inc., Cary, NC).

## Additional Information

**How to cite this article**: Yagi, M. *et al*. Antibody titres and boosting after natural malaria infection in BK-SE36 vaccine responders during a follow-up study in Uganda. *Sci. Rep.* **6**, 34363; doi: 10.1038/srep34363 (2016).

## Supplementary Material

Supplementary Information

## Figures and Tables

**Table 1:** Regression analysis for factors which may influence the geometric mean of the fold increase in antibody titre after first infection.

Explanatory variable	N	Fold increase in geometric mean and its ratio		
		Adjusted GM^a^ [95% CI]	Ratio [95% CI]	
Response to BK-SE36 vaccination (based on ≥1.92-fold increase at Day 42)				
Responder	11	3.3 [2.1, 5.3]	**2.3 [1.3, 3.8]**	1.5 [0.9, 2.6]
Non responder	26	2.2 [1.6, 3.0]	**1.5 [1.0, 2.2]**	1.0
Placebo, Control	40	1.5 [1.2, 1.9]	1.0	
p-value		**0.005**		
Age (yr)				
6–10	30	2.5 [1.9, 3.5]	1.0	
11–20	47	1.9 [1.5, 2.5]	0.8 [0.5, 1.13]	
p-value		0.175		
Anti-SE36 antibody titre before first infection				
≤52	44	2.5 [2.0, 3.2]	1.0	
>52	33	1.9 [1.4, 2.7]	0.8 [0.5, 1.1]	
p-value		0.156		
Parasitemia during the first infection (parasites/μl)				
<5,000	41	1.9 [1.4, 2.5]	1.0	
≥5,000	36	2.6 [2.0, 3.4]	1.4 [0.9, 2.0]	
p-value		0.091		

Significant results are shown in bold. N, sample size; GM, geometric mean; CI, confidence interval.

^a^All variables shown in the table are adjusted simultaneously using the multivariate linear regression. The p-value refers to the significance of the effect of the regression variable in the model.

**Table 2:** Factors which may influence risk for reinfection in volunteers.

Explanatory variable	N	n (%)	Odds ratio of each factor for reinfection			
			Crude	[95% CI]	Adjusted^a^	[95% CI]
Response to BK-SE36 vaccination (based on ≥1.92-fold increase at Day 42)						
Responder	11	2 (18)	**0.2**	[**0.03, 0.8**]	**0.1**	[**0.01, 0.5**]
Non responder	26	12 (46)	0.7	[0.3, 1.9]	0.7	[0.3, 2.2]
Placebo, Control	40	22 (55)	1.0		1.0	
Age (yr)						
6–10	30	17 (57)	1.0		1.0	
11–20	47	19 (40)	0.5	[0.2, 1.3]	0.3	[0.1, 1.03]
Anti-SE36 antibody titre after first infection (based on median arbitrary unit)						
≤80.0	39	17 (44)	1.0		1.0	
>80.0	38	19 (50)	1.3	[0.5, 3.2]	1.9	[0.7, 5.5]
Parasitemia during the first infection (parasites/μl)						
<5,000	41	18 (44)	1.0		1.0	
≥5,000	36	18 (50)	1.3	[0.5, 3.2]	1.1	[0.4, 3.1]

Significant results are shown in bold. N, sample size; n (%), frequency of volunteers with >1 infection during 130–365 days post-second vaccination; CI, confidence interval.

^a^All variables shown in the table are adjusted simultaneously using the multivariate logistic regression.

## References

[b1] Bhatt S. . The effect of malaria control on *Plasmodium falciparum* in Africa between 2000 and 2015. Nature 526, 207–211 (2015). 26375008 10.1038/nature15535 PMC4820050

[b2] Tran T. M., Portugal S., Draper S. J. & Crompton P. D. Malaria vaccines: moving forward after encouraging first steps. Curr. Trop. Med. Rep. 2, 1–3 (2015). 25995985 10.1007/s40475-015-0041-3 PMC4435961

[b3] RTS, S Clinical Trials Partnership. First results of phase 3 trial of RTS, S/AS01 malaria vaccine in African children. N. Engl. J. Med. 365, 1863–1875 (2011). 22007715 10.1056/NEJMoa1102287

[b4] RTS, S Clinical Trials Partnership. A phase 3 trial of RTS, S/AS01 malaria vaccine in African infants. N. Engl. J. Med. 367, 2284–2295 (2012). 23136909 10.1056/NEJMoa1208394 PMC10915853

[b5] RTS, S Clinical Trials Partnership. Efficacy and safety of RTS, S/AS01 malaria vaccine with or without a booster dose in infants and children in Africa: final results of a phase 3, individually randomised, controlled trial. Lancet 386, 31–45 (2015). 25913272 10.1016/S0140-6736(15)60721-8 PMC5626001

[b6] Neafsey D. E. . Genetic diversity and protective efficacy of the RTS, S/AS01 malaria vaccine. N. Engl. J. Med. 373, 2025–2037 (2015). 26488565 10.1056/NEJMoa1505819 PMC4762279

[b7] [No authors listed]. Malaria vaccine: WHO position paper-January 2016. *Wkly. Epidemiol. Rec.* **91,** 33–51 (2016). 26829826

[b8] Miura K. Progress and prospects for blood-stage malaria vaccines. Expert Rev. Vaccines. 15, 765–781 (2016). 26760062 10.1586/14760584.2016.1141680 PMC4915341

[b9] Draper S. J. . Recent advances in recombinant protein-based malaria vaccines. Vaccine 33, 7433–7443 (2015). 26458807 10.1016/j.vaccine.2015.09.093 PMC4687528

[b10] Beeson J. G. . Merozoite surface proteins in red blood cell invasion, immunity and vaccines against malaria. FEMS Microbiol. Rev. 40, 343–372 (2016). 26833236 10.1093/femsre/fuw001 PMC4852283

[b11] Thera M. A. . A field trial to assess a blood-stage malaria vaccine. N. Engl. J. Med. 365, 1004–1013 (2011). 21916638 10.1056/NEJMoa1008115 PMC3242358

[b12] Sirima S. B., Cousens S. & Druilhe P. Protection against malaria by MSP3 candidate vaccine. N. Engl. J. Med. 365, 1062–1064 (2011). 21916656 10.1056/NEJMc1100670

[b13] Sirima S. B. . A phase 2b randomized, controlled trial of the efficacy of the GMZ2 malaria vaccine in African children. Vaccine 34, 4536–4542 (2016). 27477844 10.1016/j.vaccine.2016.07.041

[b14] Horii T. . Evidences of protection against blood-stage infection of *Plasmodium falciparum* by the novel protein vaccine SE36. Parasitol. Int. 59, 380–386 (2010). 20493274 10.1016/j.parint.2010.05.002

[b15] Palacpac N. M. Q., Arisue N., Tougan T., Ishii K. J. & Horii T. *Plasmodium falciparum* serine repeat antigen 5 (SE36) as a malaria vaccine candidate. Vaccine 29, 5837–5845 (2011). 21718740 10.1016/j.vaccine.2011.06.052

[b16] Pang X. L. & Horii T. Complement-mediated killing of *Plasmodium falciparum* erythrocytic schizont with antibodies to the recombinant serine repeat antigen (SERA). Vaccine 16, 1299–1305 (1998). 9682394 10.1016/s0264-410x(98)00057-7

[b17] Pang X. L., Mitamura T. & Horii T. Antibodies reactive with the N-terminal domain of *Plasmodium falciparum* serine repeat antigen inhibit cell proliferation by agglutinating merozoites and schizonts. Infect. Immun. 67, 1821–1827 (1999). 10085023 10.1128/iai.67.4.1821-1827.1999 PMC96533

[b18] Soe S., Singh S., Camus D., Horii T. & Druilhe P. *Plasmodium falciparum* serine repeat protein, a new target of monocyte-dependent antibody-mediated parasite killing. Infect. Immun. 70, 7182–7184 (2002). 12438408 10.1128/IAI.70.12.7182-7184.2002 PMC133104

[b19] Yagi M. . Protective epitopes of the *Plasmodium falciparum* SERA5 malaria vaccine reside in intrinsically unstructured N-terminal repetitive sequences. PLoS ONE. 9, e98460 (2014). 24886718 10.1371/journal.pone.0098460 PMC4041889

[b20] Okech B. A. . Natural human immunoglobulin G subclass responses to *Plasmodium falciparum* serine repeat antigen in Uganda. Am. J. Trop. Med. Hyg. 65, 912–917 (2001). 11791998 10.4269/ajtmh.2001.65.912

[b21] Okech B. . High titres of IgG antibodies against *Plasmodium falciparum* serine repeat antigen 5 (SERA5) are associated with protection against severe malaria in Ugandan children. Am. J. Trop. Med. Hyg. 74, 191–197 (2006). 16474069

[b22] Tanabe K. . Geographic differentiation of polymorphism in the *Plasmodium falciparum* malaria vaccine candidate gene SERA5. Vaccine 30, 1583–1593 (2012). 22230587 10.1016/j.vaccine.2011.12.124

[b23] Palacpac N. M. Q. . Phase 1b randomized trial and follow-up study in Uganda of the blood-stage malaria vaccine candidate BK-SE36. PLoS ONE 8, e64073 (2013). 23724021 10.1371/journal.pone.0064073 PMC3665850

[b24] Sirima S. B. . Safety and immunogenicity of the *Plasmodium falciparum* merozoite surface protein-3 long synthetic peptide (MSP3-LSP) malaria vaccine in healthy, semi-immune adult males in Burkina Faso, West Africa. Vaccine 25, 2723–2732 (2007). 17280744 10.1016/j.vaccine.2006.05.090

[b25] Sirima S. B. . Safety and immunogenicity of the malaria vaccine candidate MSP3 long synthetic peptide in 12–24 months-old Burkinabe children. PLoS ONE 4, e7549 (2009). 19855847 10.1371/journal.pone.0007549 PMC2764341

[b26] WHO. New Perspectives: Malaria Diagnosis. Report of a Joint WHO/USAID Informal Consultation 25–27 October 1999 (WHO, Geneva, 2000) Available at: http://www.who.int/tdr/publications/documents/malaria-diagnosis.pdf (Accessed: 5^th^ August 2016).

[b27] Moody A. Rapid diagnostic tests for malaria parasites. Clin. Microbiol. Rev. 15, 66–78 (2002). 11781267 10.1128/CMR.15.1.66-78.2002 PMC118060

[b28] White M. T. . The relationship between RTS, S vaccine-induced antibodies, CD4^+^ T cell responses and protection against *Plasmodium falciparum* infection. PLoS One. 8, e61395 (2013). 23613845 10.1371/journal.pone.0061395 PMC3628884

[b29] Bell D., Wongsrichanalai C. & Barnwell J. W. Ensuring quality and access for malaria diagnosis: how can it be achieved? Nat. Rev. Microbiol. 4, 682–695 (2006). 16912713 10.1038/nrmicro1474

