# Supplementary material for: Antibody titres and boosting after natural malaria infection in BK-SE36 vaccine responders during a follow-up study in Uganda
Source: Sci Rep. 2016 Oct 5;6:34363. doi: 10.1038/srep34363 (PMC5050508; doi:10.1038/srep34363)
Supplement: Supplementary Information [file srep34363-s1.pdf]

Supplementary Information

Antibody titres and boosting after natural malaria infection in BK-SE36 vaccine responders during a follow-up study in Uganda

Masanori Yagi[1,2,a], Nirianne M. Q. Palacpac[1,a], Kazuya Ito[3,4,a], Yuko Oishi[1], Sawako Itagaki[1], Betty Balikagala[5,6], Edward H. Ntege[5,6], AdokeYeka[5,7], Bernard N. Kanoi[5,6], Osbert Katuro[5], Hiroki Shirai[8], Wakaba Fukushima[3], Yoshio Hirota[3], Thomas G. Egwang[5] & Toshihiro Horii[1*]

[1]Department of Molecular Protozoology, Research Institute for Microbial Diseases, Osaka University, 3-1 Yamadaoka, Suita, Osaka 565-0871 Japan

[2]Current address: AbbVie GK, 3-5-27 Mita, Minato-ku, Tokyo 108-6302 Japan

[3]Department of Public Health, Faculty of Medicine, Osaka City University, Osaka 545-8585, Japan

[4]Sumida Hospital, Medical Co. Living Together Association (LTA) Clinical Pharmacology Center, Tokyo 130-0021 Japan

[5]Med Biotech Laboratories, Plot 4-6 Bell Close, Port Bell Road Luzira, Kampala, Uganda

[6]Current address: Division of Malaria Research, Proteo-Science Center, Ehime University, 3 Bunkyo-cho, Matsuyama, Ehime 790-8577 Japan

[7]Department of Disease Control and Environmental Health, School of Public Health, College of Health Sciences, Makerere University, P.O. Box 7072, Kampala, Uganda

[8]The Research Foundation for Microbial Diseases of Osaka University, 2-9-41 Yahata-cho, Kanonji, Kagawa 768-0061 Japan

Correspondence and requests for materials should be addressed to T.H.

(email: horii@biken.osaka-u.ac.jp)

[a]These authors contributed equally to this work

Supplementary Table S1. Studies on boosting of antibody response to *P. falciparum* candidate vaccine antigens after natural infection.

Study	Description	Antibody dynamics	Ref
Longitudinal cohort (Gambia)	Children under 7 years of age<br>(n=34, with clinical malaria;<br>n=69, without clinical malaria);<br>IgG profiles of AMA1, EBA175, $MSP1_{19}$, MSP2 and crude schizont extract in relation to clinical malaria over a 10-month period<br>(dry to wet season:<br>Day 0, dry season;<br>Day 154, beginning of wet season;<br>Day 285, end of malaria transmission season)	Children with clinical malaria had lower antibody levels at the start of the study than those who did not experienced clinical malaria.<br><br>Serum IgG antibody levels to merozoite antigens increased during the wet season in children with clinical malaria (the antibody levels were significantly higher at D285 than D0 or D154). In contrast, increase in antibody levels were seen only in 1/3 of those who did not have clinical malaria.<br><br>Peak antibody levels occurred during or immediately following clinical infection and gradually declined following treatment and resolution.	[1]
Nested case control study (Thailand)	Pregnant women<br>(n=136, with infection;<br>n=331, uninfected controls).<br>Weekly samples for blood smear and fortnightly for serum samples throughout pregnancy until delivery.<br>Antibody responses to merozoite antigens of *P. falciparum*<br>(AMA1, EBA175, MSP2, MSP3, schizont extract) and *P. vivax* (AMA1) and pregnancy specific PfVAR2CSA	Women could be broadly classified into having dynamic or relatively stable IgG antibody responses.<br><br>Concurrent infection in pregnant women has a role in boosting or maintaining antibody responses. Antibody levels to PfVAR2CSA and Pf merozoite antigens showed evidence of boosting with each successive *P. falciparum* infection.<br><br>Antibodies to merozoite antigens were relatively short-lived compared with antibodies for other pathogens (e.g. measles). However, antibodies to PfVAR2CSA persist much longer and are maintained throughout pregnancy.<br><br>Generally, antibodies are not maintained at a constant level, but fluctuate around a threshold, even in the absence of parasite infection.	[2]
Prospective cross sectional study (Uganda)	n=362 malaria patients, 6 months to 60 years-old in 3 cohorts (<5 years, 6-15 and >16 years-old)<br>Monitoring of IgG antibodies to GLURP, MSP3 and HRPII peptides at infection but	42 days after malaria diagnosis and initiation of Coartem treatment, majority of patients (70%) developed at least 20% increase in level of anti-parasite IgG<br><br>The increase in level of IgG antibody (GLURP and MSP3 specific antibodies) was not influenced by parasite density on the day of	[3]

	before artemether-lumefantrine treatment (Day 0) and on Day 42 (after treatment)	malaria diagnosis or by HIV status<br><br>Fold increase in anti-parasite IgG was not significantly different among the 3 age cohorts.	
Longitudinal study (Mali)	Children (2-10 years, n=176) and adults (18-25 years, n=49). Blood smear and venous sample collected during 2-week period before and at the end of the 6-month malaria season. Microarray to 1,204 Pf proteins	Gametocyte specific IgG responses increased during the malaria season (IgG responses to Pfs48/45 and Pfs230 could be boosted but not Pfs25).<br><br>Boosting was observed in children (2-5 years, 6-10 years) but not in adults (18-25 years-old).<br><br>Seroprevalence of both gametocyte specific and non-gametocyte specific IgG increased from before to after malaria season (p<0.0001).	[4]
Interventional Phase Ia/IIa (UK adults)	Antibody responses against both MSP1 and AMA1 following ChAd63-MVA immunization and controlled human malaria infection (CHMI)	Serum IgG responses against both MSP1 and AMA2 in vaccinees increased post CHMI infection and drug treatment (2-fold increase by 35 days post CHMI)<br><br>Immune response was strongly associated with level of parasitemia at diagnosis<br><br>Pre-existing immune responses (to AMA1) may aid the induction of *de novo* responses against other blood-stage antigens ($MSP1_{19}$)	[5]
Interventional Phase Ib (Burkina Faso)	Adult males (18-40 years) Vaccination: Day0, 28, 112 Antibody response to MSP3-LSP on days 0, 28, 56, 112, 140, 252 and 365 of the trial	In vaccine group, no major increase in anti-MSP3 antibody levels during the malaria transmission season (Day 140-252)	[6]

[1] Akpogheneta, O.J., Dunyo, S., Pinder, M. & Conway, D.J. Boosting antibody responses to *Plasmodium falciparum* merozoite antigens in children with highly seasonal exposure to infection. *Parasite Immunol.* **32,** 296–304 (2010).

[2] Fowkes, F.J, *et al.* New insights into acquisition, boosting, and longevity of immunity to malaria in pregnant women. *J. Infect. Dis.* **206,** 1612-1621 (2012).

[3] Kaddumukasa, M. *et al.* Parasite specific antibody increase induced by an episode of acute *P. falciparum* uncomplicated malaria. *PLoS One* **10,** e0124297 (2015).

[4] Skinner, J. *et al. Plasmodium falciparum* gametocyte-specific antibody profiling reveals boosting through natural infection and identifies potential markers of gametocyte exposure. *Infect. Immun.* **83,** 4229-4236 (2015).

[5] Biswas, S. *et al.* Assessment of humoral immune responses to blood-stage malaria antigens following ChAd63-MVA immunization, controlled human malaria infection and natural exposure. *PLoS One* **9,** e107903 (2014).

[6] Sirima, S.B. *et al.* Safety and immunogenicity of the *Plasmodium falciparum* merozoite surface protein-3 long synthetic peptide (MSP3-LSP) malaria vaccine in healthy, semi-immune adult males in Burkina Faso, West Africa. *Vaccine* **25,** 2723-2732 (2007).

Supplementary Table S2. Factors which may influence fold increase in antibody titer after first infection.

Explanatory variable	N	n (%)	Odds ratio Crude	[95% CI]	Adjusted[a]	[95% CI]
Response to BK-SE36 vaccination (based on ≥ 1.92-fold increase at Day 42)						
Responder	11	7 (64)	**15.7**	**[3.4, 89.0]**	**18.2**	**[3.3, 128]**
Non responder	26	8 (31)	**4.0**	**[1.1, 16.7]**	**5.5**	**[1.4, 25.3]**
Placebo, Control	40	4 (10)	1.0		1.0	
Age (yr)						
6-10	30	10 (33)	1.0		1.0	
11-20	47	9 (19)	0.5	[0.2, 1.4]	0.8	[0.2, 3.7]
Anti-SE36 antibody titer before first infection						
≤ 52	44	13 (30)	1.0		1.0	
> 52	33	6 (18)	0.5	[0.2, 1.5]	1.4	[0.3, 6.2]
Parasitemia during the first infection (parasites /μl)						
< 5,000	41	7 (17)	1.0		1.0	
≥ 5,000	36	12 (33)	2.4	[0.9, 7.4]	2.9	[0.7, 13.4]

Significant results are shown in bold. N, sample size; n (%), frequency of volunteers with >3.35-fold increase in antibody titer; CI, confidence interval. [a]All variables shown in the table are adjusted simultaneously using the multivariate logistic regression.

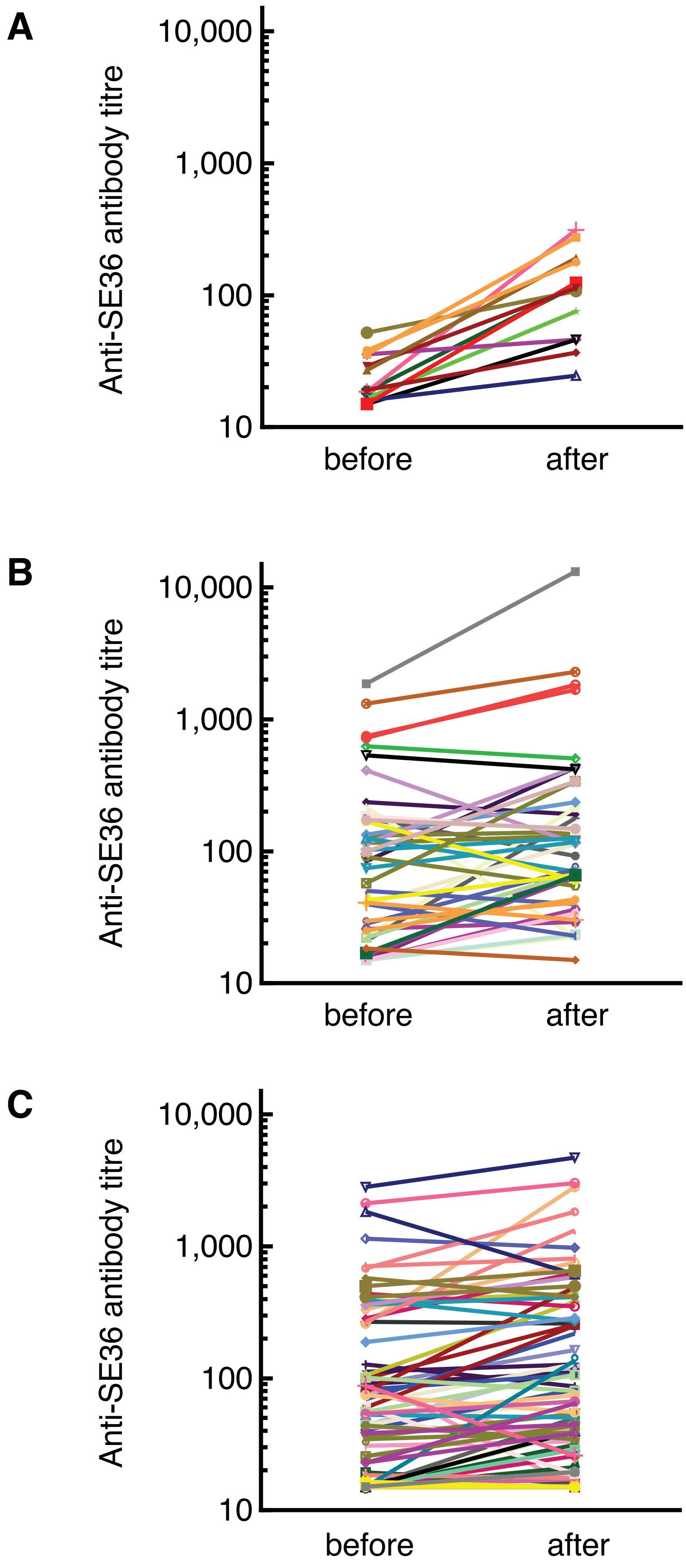

Figure S1. Anti-SE36 antibody titre dynamics before and after natural infection in the responder (**A**), non responder (**B**) and placebo/control (**C**). In each group, one color represent one child.
